# Evaluation of epicardial adipose tissue in familial partial lipodystrophy

**DOI:** 10.1186/s13098-015-0024-5

**Published:** 2015-04-01

**Authors:** Amélio Fernando Godoy-Matos, Cynthia M Valério, Juliana Bonadiman e Bragança, Ricardo de Andrade Oliveira, Roberto Luis Zagury, Rodolfo de Paula Lustosa, Gabriel Cordeiro Camargo, César Augusto da Silva Nascimento, Rodrigo O Moreira

**Affiliations:** Metabolism Unit, State Institute of Diabetes and Endocrinology, IEDE, Rio de Janeiro, Brazil; National Cardiology Institute of Laranjeiras, Rio de Janeiro, Brazil

**Keywords:** Lipodystrophy, Epicardial adipose tissue, Echocardiography, Metabolic syndrome, Dual energy x-ray absorptiometry

## Abstract

**Background:**

Dunnigan type Familial Partial Lipodystrophy (FPLD) is characterized by loss of subcutaneous fat from the limbs and excessive accumulation on the visceral adipose tissue (VAT). Affected individuals have insulin resistance (IR), diabetes, dyslipidemia and early cardiovascular (CV) events, due to their imbalanced distribution of total body fat (TBF). Epicardial adipose tissue (EAT) is correlated with VAT. Hence, EAT could be a new index of cardiac and visceral adiposity with great potential as a marker of CV risk in FPLD.

**Objective:**

Compare EAT in FPLD patients versus healthy controls. Moreover, we aimed to verify if EFT is related to anthropometrical (ATPM) and Dual-Energy X-ray Absorptiometry (DEXA) measures, as well as laboratory blood findings. We postulated that FPLD patients have enlarged EAT.

**Methods:**

This is an observational, cross-sectional study. Six patients with a confirmed mutation in the ***LMNA*** gene for FPLD were enrolled in the study. Six sex, age and BMI-matched healthy controls were also selected. EFT was measured by transthoracic echocardiography (ECHO). All participants had body fat distribution evaluated by ATPM and by DEXA measures. Fasting blood samples were obtained for biochemical profiles and also for leptin measurements.

**Results:**

Median EFT was significantly higher in the FPLD group than in matched controls (6.0 ± 3.6 mm vs. 0.0 ± 2.04 mm; p = 0.0306). Additionally, FPLD patients had lower leptin values. There was no significant correlation between EAT and ATPM and DEXA measurements, nor laboratory findings.

**Conclusions:**

This study demonstrates, for the first time, that EAT measured by ECHO is increased in FPLD patients, compared to healthy controls. However, it failed to prove a significant relation neither between EAT and DEXA, ATPM or laboratory variables analyzed.

## Introduction

Lipodystrophies (LD) are clinically heterogeneous acquired or inherited disorders characterized by generalized or partial loss of adipose tissue [1]. Familial Partial Lipodystrophy, Dunnigan variety (FPLD) is a rare autosomal dominant disorder due to missense mutations in the lamin A/C gene. It is phenotypically characterized by the gradual loss of subcutaneous (SC) fat from the extremities and trunk, starting in puberty, with a selective visceral lipodeposition and fat accumulation in shoulder girdle, neck and face [1-3].

In spite of being a rare condition, the better understanding of this underexplored pathology can reveal important clues to deciphering insulin resistance (IR) and its metabolic consequences. FPLD patients have marked IR with glucose intolerance (GI) or diabetes (DM), dyslipidemia, hepatic steatosis (HS), acanthosis nigricans and a high risk of cardiovascular (CV) disease. Such risk is similar to that described for visceral obesity, which is associated with the development of metabolic syndrome (MS) and its increased CV morbidity and mortality [2,3].

Determining the patterns of adipose tissue distribution is, thus, a crucial matter for FPLD patients, as for obese individuals [2,4]. Fat depot quantification has been studied in FPLD [2,4-6]. Anthropometric (ATPM) methods are limited in their ability to evaluate sites and extent of fat deposition. However, waist circumference is widely accepted as a good predictor of VAT. Radiologic methods like dual-energy x-ray absorptiometry (DEXA), computed tomography (CT) and magnetic resonance imaging (MRI) have been proved to be better tools to accurately measure body fat depots. Unfortunately, CT requires radiation exposure and, like MRI, is quite a high-cost method [2,4]. This later appears to be a reliable method and have also been validated in FPLD, using both qualitative and quantitative analysis [4-6]. A volumetric and single-slice MRI adipose analysis using a novel fully automated segmentation method has been also recently validated [7]. However, the high-cost limits its use. Several studies have reported that the ECHO- assessed epicardial fat tissue (EFT) highly correlates with VAT determined by MRI [8-10]. EAT shares a common embryological origin with mesenteric and omental fat. It might function as a lipid-storing depot, as an endocrine organ secreting hormones, and as an inflammatory tissue secreting cytokines and chemokines. Like VAT, ECHO-measured EFT is increased in visceral obesity. Recent studies are testing the hypothesis that human EFT might contribute locally to the pathogenesis of coronary atherosclerosis [10-13]. EFT can be visualized and measured by transthoracic echocardiography, as first proposed and validated by Iacobellis et al. [8]. Although radiologic techniques can provide a volumetric and more accurate measurement, echocardiographic assessment of EFT certainly has the advantages of being inexpensive, noninvasive, and readily available, accurate and reproducible method [8,9,11]. ECHO measurement of EFT has been reported in HIV-Infected patients with highly active antiretroviral therapy (HAART)-associated MS and LPD [14]. It has also been validated in obese and MS patients [8,11,15]. A recent meta-analysis evaluated the relation between EAT and MS, and concluded that EAT thickness is significantly higher in patients with MS (standardized difference in means 1.15, 95% confidence interval 0.78 to 1.53, p = 0.0001) [16]. Fernandez-Muñoz et al., have tested the hypothesis that EFT is associated with VAT, MS, and insulin resistance in postmenopausal women. They showed that EFT has a strong correlation with BMI, WC, and VAT, as well as with the presence of MS [17]. The present study is the first evaluating the EFT thickness in FLPD subjects.

In this setting, the primary objective of the present study was to provide original information about ECHO-derived EFT measurement in FPLD subjects and to assess its correlation with simple ATPM and DEXA measures. We postulate that FPLD patients have enlarged EFT measures, compared to their matched controls, in agreement to ATPM and DEXA parameters.

Our secondary aim was to evaluate whether EFT measures obtained by ECHO are related to leptin values, as well as to glucose and lipid profiles, in affected subjects.

## Materials and methods

This is an observational, cross-sectional study held in the Metabolism Department, an outpatient facility of the State Institute of Diabetes and Endocrinology, IEDE (Rio de Janeiro, Brazil). It was approved by our local ethics committee and all subjects provided written, informed consent.

### Genetic assays

Mutational analysis of *LMNA* gene was carried out in 12 female patients with lipodystrophy phenotype, selected from an outpatient facility. In six of them, mutations known to be associated with FPLD were found. Three patients were heterozygote for the FPLD mutation *LMNA* R482W and the other three patients had FPLD mutation *LMNA* R482Q. Hence, six patients who had genetically confirmed FPLD were selected for this study.

Exclusion criteria adopted were: not to be pregnant or breast feeding; age <18 years; not to have acquired LPD (auto-immune, AIDS or HAART linked); severe renal or hepatic diseases; depression or alcoholism. Recent use of thiazolidinediones (TZD), glucocorticoids, or significant weight loss (≥3 Kg, in the last 3 months), were other exclusion criteria.

Control volunteers were recruited and were age, gender, and body mass index (BMI)-matched with the FPLD subjects. All controls were healthy and had no previous medical conditions likely to influence the evaluation at the time of the study.

### Anthropometry and body composition

ATPM and body composition measurements were performed in all study participants, with the subject wearing light clothing without shoes. For all subjects, weight and height were measured to the nearest 0.5 kg and 0.5 cm, respectively, and the BMI was calculated as weight (kilograms) divided by height squared (square meters). Waist circumference (WC) and hip circumference were also measured by the same observer, and the waist-to-hip ratio (WHR) was calculated.

### Biochemical measurements

Blood samples were collected from all subjects after an overnight fast (12 hours) between 0630 h and 0800 h. Plasma glucose was determined by the glucose-oxidase method. Cholesterol contents of lipoproteins fractions and triglycerides were measured enzymatically. Plasma leptin and insulin concentrations were measured by radioimmunoassay. Fasting and 2-h postload glucose (G 2 h) levels after 75-g oral glucose tolerance test (OGTT) were determined. Insulin resistance (IR) was estimated through the homeostasis model assessment (HOMA-IR), using the formula: IR = fasting insulin × fasting glucose/22.5.

### Dual-energy x-ray absorptiometry

Whole body fat assessment by DEXA was performed using GE Lunar Prodigy Advance software, version 9.5, LNR 41569 model (GE Lunar Corp., Madison, WI),to determine fat and lean soft-tissue mass in the trunk and legs, in FPLD patients and their matched normal healthy controls. The android/gynoid ratio [A/G R] and central to peripheral fat Ratio (or Fat Mass Ratio [FMR]) was used to investigate body fat distribution [18].

### Echocardiographic Study

Each subject underwent transthoracic two-dimensional guided M-mode ECHO. ECHO was performed with a Siemens SONOLINE Omnia instrument (Issaquah, WA), by standard techniques, with subjects in the left lateral decubitus position. ECHO were preliminarily read by a first reader and recorded for future revaluation. Subsequently they were reread by another experienced reader. Both readers were blinded to subjects’ anthropometric features. The EFT appears as an echo-free space and was measured on the free wall of the right ventricle from both parasternal long- and short-axis views, as previously described by Iacobellis et al. [8,14].

### Statistical analysis

Analysis was undertaken using BioEstat (Belém, PA) and p-value less than 0.05 was considered statistically significant for all analyses. All variables were expressed as continuous. Descriptive statistics are presented as the median and standard error in tables. Non-parametric tests were performed, because of the non-Gaussian distribution of the variables, data dispersion and the lack of symmetry of the reduced size sample.

Comparison between the FPLD *vs.* the control group was provided by Mann-Whitney tests. The variable interactions were tested for significance with Spearman tests, as follows: between EFT and each of the DEXA parameters; EFT and BMI, waist-to-hip ratio (WHR) and age, as well as with EFT and laboratorial findings (leptin, fasting glucose [FG], 2hG, total cholesterol [TC], HDL, LDL, VLDL and HOMA-IR).

## Results

Table 1 summarizes ECHO, biochemical, ATPM and DEXA measures of the 12 female patients enrolled in this study. There was no significant difference in age (p = 0.87) or BMI (p > 0.05) between FPLD patients and controls. Median EFT was significantly increased in FPLD subjects (p = 0.0306).

**Table 1:** **Descriptive and comparative anthropometrical, echocardiography, DEXA and laboratory findings of FPLD group vs. control group**

	**FPLD (min-max)**	**CONTR (min-max)**	**P-VALUE**
**AGE (years)**	36.95 (27.9-46.6)	35.45 (28.3-48.5)	0.8728
**BMI (kg/m2)**	22.85 (20.5-27.6)	22.05 (21.3-24)	0.3367
**WHR**	0.95 (0.8-1.04)	0.76 (0.7-0.87)	0.0163
**EFT (mm)**	6.0 (0-8)	0.0 (0-5)	0.0306
**TBF (%)**	17.4 (16.1-27.6)	39.15 (32.2-45.2)	0.0039
**FMR**	1.17 (0.70-2.91)	0.64 (0.47-0.82)	0.0104
**A/G R**	1.19 (0.8-1.49)	0.82 (0.67-1.06)	0.0163
**FG (mg/dl)**	90 (75-115)	83.5 (74-98)	0.298
**G2h (mg/dl)**	117 (108-213)	99.5 (74-134)	0.1735
**INS (pmol/L)**	10.7 (4.8-42.8)	6.6 (2-15.8)	0.2623
**HOMA-IR**	2.46 (0.91-12.14)	1.25 (0.36-3.81)	0.2002
**LEPTIN (ng/ml)**	4.4 (2.4-7.3)	16.75 (6.1-30.1)	0.0131
**TC (mg/dl)**	213 (186-264)	218 (156-283)	0.631
**LDL (mg/dl)**	139.5 (102-163)	118.5 (82-207)	0.5218
**HDL (mg/dl)**	39.5 (29-43)	65 (46-91)	0.0039
**TG (mg/dl)**	203.5 (129-321)	110 (52-212)	0.0782

Abbreviations: familial partial lipodystrophy, Dunnigan type group [FPLD]; Dual-Energy X-ray Absorptiometry [DEXA]; control group [CONTR]; epicardial fat tissue [EFT]; p-value [p]; body mass index [BMI]; waist-to-hip ratio [WHR]; total body fat [TBF%]; android/ginecoid ratio [A/G R]; fat mass ratio [FMR], fasting glucose [FG]; 2-h post load glucose [G2h]; total cholesterol [TC]; LDL- cholesterol [LDL]; HDL-cholesterol [HDL]; VLDL-cholesterol [VLDL]; triglycerides [TG].

FPLD patients also had ATPM and DEXA markers of increased central abnormal fat accumulation and less peripheral fat, noticeably by higher WHR (p = 0.0163) and android/ginoid ratio [A/G R] (p = 0.0163), as well as by decreased total body fat [TBF%] (p = 0.0039) and increased fat mass ratio [FMR] (p = 0.0104).

The biochemical parameters are also displayed in Table 1. As for laboratory findings, we observed that HDL-cholesterol (p = 0.0039) and leptin (p = 0.0131) were expressively lower in the FPLD group than in the control group. These laboratorial findings reinforce the unfavorable metabolic profile, related to the impaired peripheral fat distribution patterns, in FPLD patients, as previously reported in the literature.

Observing that FPLD patients had increased EFT, in agreeance to DEXA and ATPM parameters, as expected, one could propose a likely relation between EFT and ATPM and DEXA. However, even though merely descriptive and comparative data would suggest such a statement, it was refuted by Spearman Correlation tests, as shown in Table 2.

**Table 2:** **Spearman Coefficient (r**_**s**_ **) relations between Epicardial Fat Tissue (EFT) and anthropometric (Age, BMI and WHR), DEXA (TBF, A/G R and FMR) and laboratory variables**

	**Age**	**BMI**	**WHR**	**TBF%**	**A/G R**	**FMR**	**FG**
**rs**	0.56	0.18	0.68	0.29	0.68	0.03	0.44
**p**	0.25	0.73	0.14	0.57	0.14	0.96	0.38
	**G2h**	**Insulin**	**TC**	**LDL**	**HDL**	**TG**	**Leptin**
**rs**	0.07	0.0003	0.15	0.24	−0.44	−0.26	0.60
**p**	0.89	ns	0.78	0.65	0.38	0.61	0.21

Abbreviations: epicardial fat tissue [EFT]; Spearman coefficient [r_s_]; p-value [p]; body mass index [BMI]; waist-to-hip ratio [WHR]; total body fat [TBF%]; android/ginecoid ratio [A/G R]; fat mass ratio [FMR]; fasting glucose [FG]; 2-h post load glucose [G 2h]; total cholesterol [TC]; LDL- cholesterol [LDL]; HDL-cholesterol [HDL]; triglycerides [TG].

## Discussion

Although several reports have been performed evaluating ectopic fat depots in patients with FPLD and LMNA mutations, this study was the first to test the hypothesis that EFT thickness is increased in FPLD patients [19,20]. This observation is in accordance to previous findings in other conditions in literature, such as central obesity, coronary artery disease (CAD), type 2 diabetes mellitus (T2D), and MS [16,21]. Our patients had increased EFT, in agreement with prior results evaluating VAT in patients with FPLD [19,20]. Nonetheless, although our patients had increased EFT, as well as ATPM and DEXA parameters suggestive of less peripheral fat deposition, no statistical relation between these variables was found, in opposition to what we expected. We attribute the lack of significance of these relations to our limited size sample (Type 2 statistic error). Otherwise, it has been demonstrated that there is a good correlation between pericardial fat measured by echocardiography and anthropometric parameters of metabolic syndrome [21,22].

Furthermore, recent reports have suggested threshold values of EAT associated with metabolic and ATPM risk factors. Cut-off points for EAT thickness have been proposed to diagnose MS [21,22]. Okyay et al. reported that EAT thickness of > 4.35 mm indicated MS according to the International Diabetes Federation [22]. In our study, median EFT in FPLD patients was 6.0 ± 3.6 mm versus 0.0 ± 2.04 mm in matched control subjects. These results are in agreement with data recently published in MS patients in whom mean EFT thickness was 5.8 + 1.9 mm [15].

Additionally, it has been suggested that EFT thickness, an indicator of cardiac adiposity, may be significantly related to inflammatory adipocytokines (plasma visfatin and plasminogen activator inhibitor-1 levels) in visceral-obese patients, reinforcing that EFT might be used as an easy and reliable marker of VAT and inflammation and as a cardiovascular risk indicator [10,23].

Hence, EAT could be a promising marker of VAT accumulation, and ECHO could be a cost-effective technique for the diagnosis of FPLD and its treatment management. Determining EFT could also be useful for therapeutic interventions with drugs that could modulate EFT paracrine activity in the heart [24]. There are few studies evaluating drug interventions on epicardial fat. Thiazolidinediones improve fat metabolism and reduce systemic inflammation through their actions as peroxisomal proliferator activating receptor-gamma (PPAR-gama) agonists. Pioglitazone reduced the expression of interleukins in epicardial adipose tissue of MS and Type 2 Diabetes [25]. Data about epicardial fat are limited, but it has also been showed that atorvastatin reduced EAT in coronary artery disease (CAD) patients [26]. There are no studies evaluating pharmaceutical interventions on EFT in patients with FLPD. Apart the good perspectives, nevertheless, one should attempt for observer-dependent reproducibility of this technique, that could be a limitation to its widely use [27].

Regarding EFT adipocytokine production, our findings revealed a marked hypoleptinemia in FPLD patients versus their controls. Positive associations between leptin and TBF% were found, which indicates, as expected, that lower peripheral fat deposition correlates well with lower leptin values. Hypoleptinemia has been incriminated in ectopic fat deposition, lipotoxicity and hyperphagia, as well as induced increased energy expenditure in FPLD affected subjects [28]. Nevertheless, these pathological features could be ameliorated in response to leptin replacement therapy, with promising results in the lipid and glucose metabolism, through the IR reduction [29].

Interestingly, leptin can be considered one of the common denominators of IR in LD and obesity. Remarkably, leptin lies at the interface of lipodystrophy and obesity [30]. Hence, better understanding of FPLD, a natural single-gene model of MS, could be the key to unlock leptin resistance in obesity, allowing leptin to perform its insulin-sensitizing function.

It could be hypothesized that higher EAT thickness might be implicated in the pathophysiology of the MS (and maybe FPLD), because it, as an endocrine organ, produces a number of factors that influence insulin sensitivity. However, it appears more plausible to assert that higher EAT thickness may serve as an indicator of visceral fat accumulation.

In summary, we present a cross-sectional study on the fat distribution pattern of FPLD patients and demonstrate that EFT is increased in these individuals, compared to matched controls. It would be of interest to examine larger numbers of affected FPLD patients to confirm the previously observed relationship between EFT and VAT. Further detailed studies are needed to conclusively address these topics and unravel underlying mechanisms that could link FPLD to IR. In conclusion, the better understanding of FPLD could provide novel insights for interventions in MS and obese patients.

## Abbreviations

FPLD: Familial partial lipodystrophy Dunnigan type
EAT: epicardial adipose tissue
ECHO: Echocardiography
DEXA: Dual-Energy X-ray Absorptiometry
BMI: Body mass index
WHR: Waist-to-hip ratio
FMR: Fat mass ratio
HOMA-IR: Homeostasis model assessment
IR: Insulin resistance
